# Normal Pressure Hydrocephalus

**DOI:** 10.7759/cureus.35131

**Published:** 2023-02-18

**Authors:** Shaan Patel, Mekdes Ditamo, Rohan Mangal, Murdoc Gould, Latha Ganti

**Affiliations:** 1 Biology, John Burroughs School, St. Louis, USA; 2 Neurology, Orlando Health, Orlando, USA; 3 Medical School, University of Miami Miller School of Medicine, Miami, USA; 4 Biology, Rollins College, Orlando, USA; 5 Emergency Medicine, HCA Florida Ocala Hospital, Ocala, USA; 6 Emergency Medicine, Envision Physician Services, Plantation, USA; 7 Emergency Medicine, University of Central Florida College of Medicine, Orlando, USA

**Keywords:** reversible dementia, ventriculoperitoneal shunt placement, vp shunt, normal pressure hydrocephalus, nph

## Abstract

Normal Pressure Hydrocephalus (NPH) occurs when there is an accumulation of cerebrospinal fluid due to impeded flow or excess production, resulting in gait and memory impairment and urinary incontinence. The authors present the case of a 67-year-old male, who had symptoms for a year prior to being diagnosed. His neurological exam was significant for a slow, and unsteady wide-based gait. No underlying cause for his NPH was found. He underwent a shunt procedure following which he made a complete recovery.

## Introduction

Normal pressure hydrocephalus (NPH) first became recognized as a treatable, reversible disorder in the 1960s [1]. NPH occurs when there is impairment of normal flow of cerebrospinal fluid (CSF). There are four main types of hydrocephalus in adults: obstructive, communicating, hypersecretory, and normal pressure hydrocephalus [2]. NPH is characterized by urinary incontinence, gait apraxia, and memory impairment [3], the classic “wet, wobbly, and wacky.” Imaging demonstrates enlarged ventricles, and lumbar puncture will yield a normal opening pressure. The NPH score can be used to describe a patient’s limitations, with a lower score indicating a higher level of limitation [4]. 

NPH can be idiopathic, where there is no identifiable etiology, or secondary. Secondary causes of NPH include subarachnoid hemorrhage, infection, head trauma, or tumor. The estimated incidence is 5.5 per 100,000. The prevalence is estimated to be 0.41-2.94% throughout five different studies on the prevalence of idiopathic NPH in elderly patients, from different countries [5].

## Case presentation

A 67-year-old male presented due to difficulty with balance. He reported the last time he was able to walk in a straight line was approximately one year ago. At that time he was healthy and had no other symptoms. He progressively developed difficulty with his balance and noticed he had to separate his legs to be able to keep himself from falling. He had a cane and a walker and has used them for the most part of the current year. He also complained of short-term memory loss, mainly for day-to-day events, and also some occasional word-finding difficulty. He was otherwise completely independent in feeding, dressing, hygiene, and all other activities of daily living. He also reported an increased frequency of urination, explaining that on a couple of occasions, he had urinated on himself because he was not able to reach the bathroom fast enough. He was uncertain if he was able to feel a full bladder. His past medical history was significant for diabetes, hypertension, and atrial fibrillation. 

His vital signs included a temperature of 98.4 ºF, a heart rate of 72 beats per minute, a respiratory rate of 16 breaths per minute, a blood pressure of 144/82 mmHg, and pulse oximetry of 100. Physical examination revealed a slightly overweight male who appeared his stated age, who was speaking fluently and could obey commands. He was alert, oriented, and in no acute distress. His general appearance was that of one with no distress, a pleasant demeanor, conversational, with normal mental status. On formal testing, there was no evidence of visual constructional apraxia or motor apraxia. His mini mental status exam (MMSE) score was 28, with 2 points off for not being able to complete counting serial sevens, and struggling with spelling "WORLD" backward. The patient's cranial nerve exam revealed that the patient's pupils were sluggish to react to light, and there was mild horizontal gaze dysconjugation with the right eye appearing slightly esotropic, but without obvious extraocular movement restriction. All the other cranial nerves were normal. The patient's motor exam was normal. His sensory was dulled in some areas. He had a mild decrease of vibration sensation at his toes and ankles and a length-dependent decrease in pinprick and temperature sensation along the dorsal aspect of the foot and mid to distal leg medially. In addition, his Romberg sign was positive. His deep tendon reflexes were as follows: 2 in the biceps and brachioradialis tendons, 1 in the patellar tendon, and his ankle reflexes were unobtainable. In his movement, there was minimal amplitude bilateral/symmetric postural and kinetic tremor of distal upper extremities approximately 6 Hz without any resting tremor or bradykinesia. His gait was slow, unsteady with a wide-based stance, and short stride length. When standing with eyes closed, he would sway to the left. 

**Figure 1:**
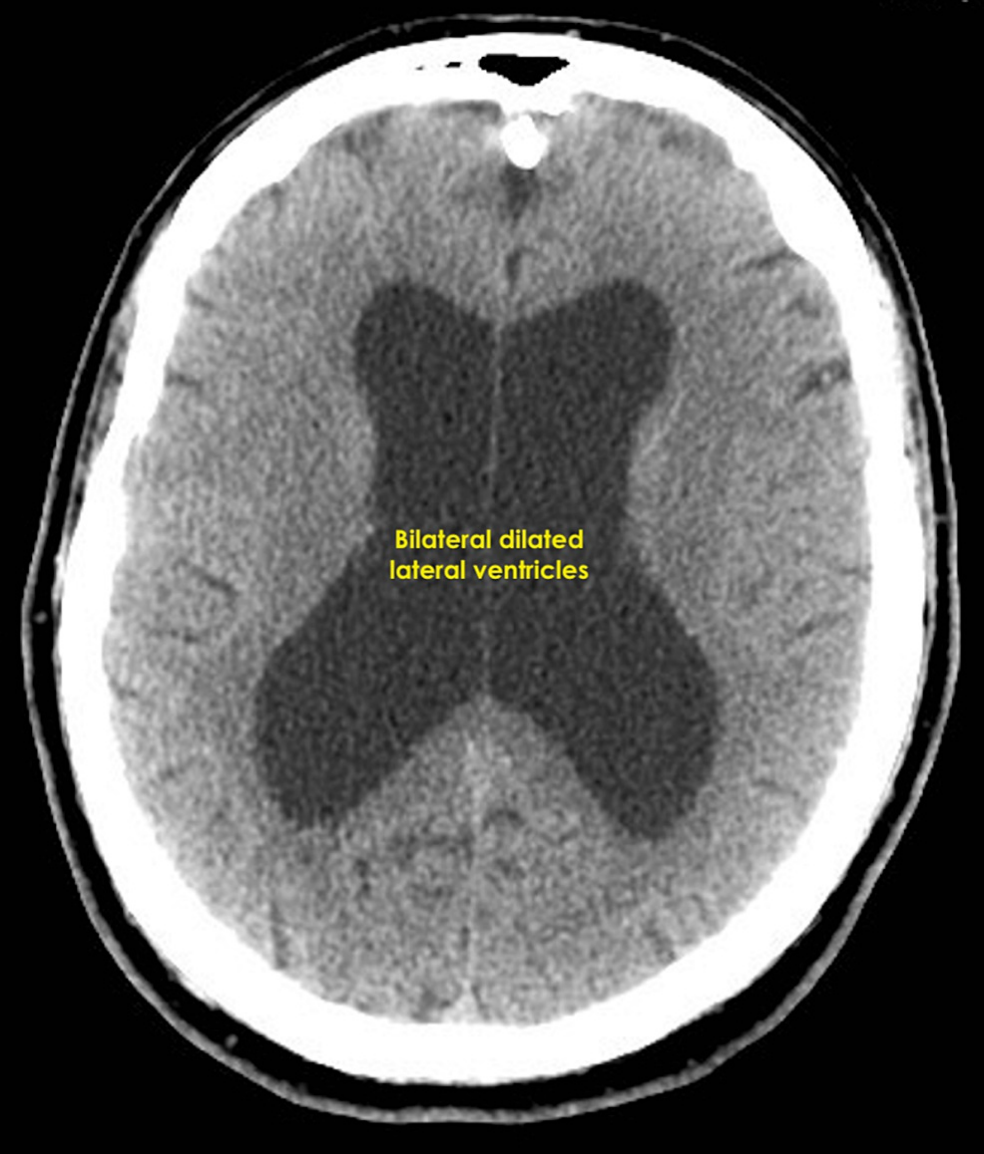
Axial brain computed tomography scan demonstrating bilateral dilated lateral ventricles typical of hydrocephalus

Noncontrast brain computed tomography demonstrated enlarged lateral ventricles. He underwent a shunt procedure to drain the cerebrospinal fluid and went on to make a significant recovery (Figure 1).

**Figure 2:**
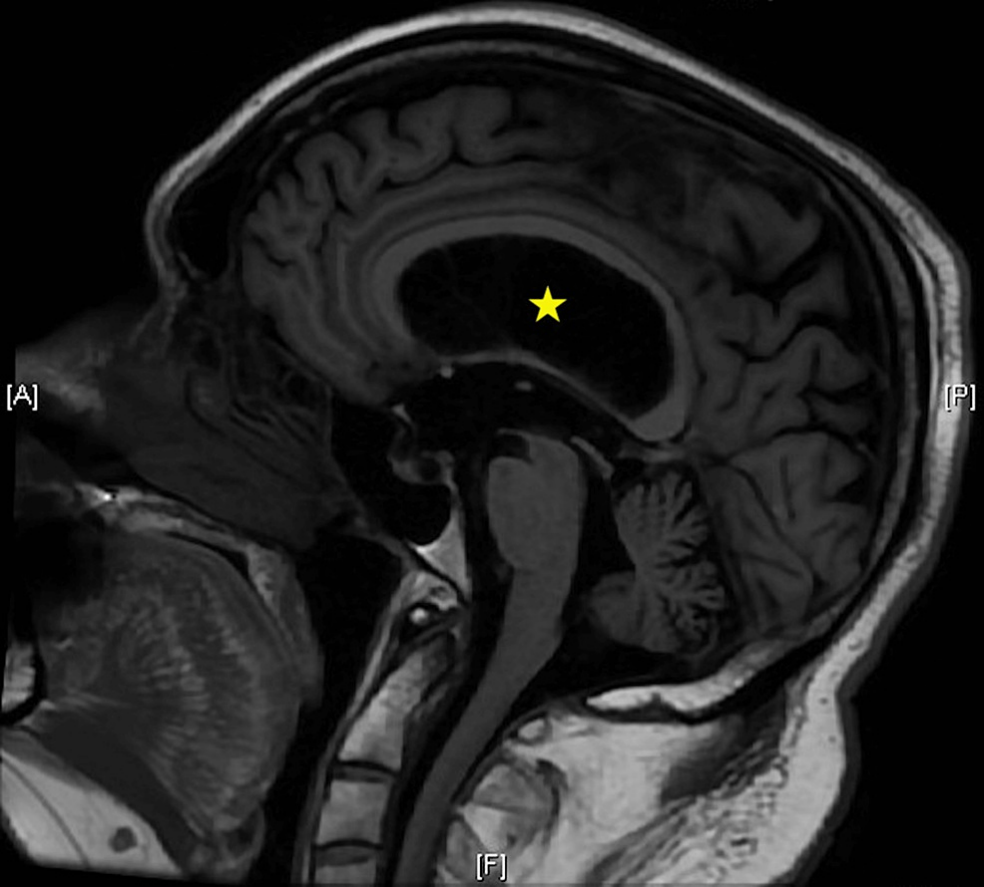
Magnetic Resonance image saggital view demonstrating interval improvement in hydrocephalus (star).

Fourteen months later, the patient was seen in follow-up, and magnetic resonance imaging demonstrated interval improvement of the hydrocephalus, with sustained improvement in gait (Figure 2). 

## Discussion

Normal pressure hydrocephalus is one of the very few reversible causes of dementia [2], therefore early diagnosis and treatment is beneficial to the patient in preventing progression of disease until point of irreversibility. The pathophysiology of NPH remains unknown. Many theories have been proposed surrounding CSF disturbance [6]. Normal CSF flow begins from the choroid plexus where CSF is made, it flows to the lateral ventricles, then through the foramen of Monro to the third ventricle, then through the aqueduct of Sylvius into the fourth ventricle, from there it exits through the foramen of Luschka or Magendie to enter the subarachnoid space where it is reabsorbed by arachnoid villi in the superior sagittal sinus [7]. CSF disturbance theory proposes disturbance in CSF flow and reabsorption, leading to brain parenchyma alteration and vascular abnormalities leading to the clinical manifestation of NPH [8]. 

NPH is believed to be underdiagnosed and undertreated [9]. Factors that could contribute to underdiagnosis and undertreatment include lack of the expected NPH triad, lack of widely accepted standardized diagnostic criteria, and clinical overlap with other neurodegenerative disorders such as parkinsonian syndromes, and Alzheimer's dementia [10].

NPH is widely known to present with disturbances in gait, bladder control, and memory, with gait disturbance being the initial presentation [1]. The diagnosis of NPH remains a challenge for the above-mentioned reasons. When there is a high index of suspicion for NPH, the CSF tap test (CSF-TT) is the only test that can temporarily simulate the effect of a definitive shunt [11]. There are several publications made in an attempt to bring more awareness of NPH and to help in a timely diagnosis. One example of such a publication is the Japanese guideline for the diagnosis and treatment of NPH, which combines clinical, radiological, and improvement of symptoms after large volume tap or temporary shunt placement [12]. This guideline uses clinical presentation and imaging studies to stratify patients into possible, probable, or definite pools, with "definite" labeling reserved for those whose symptoms resolve after CSF shunting. Advancements in diagnosis and treatment are being investigated such as using positron emission tomography (PET) scans to differentiate NPH from other neurodegenerative disorders [13] as well as measuring CSF flow parameters to predict the outcome of definite shunt placement [14].

NPH is treated with CSF diversion, which includes ventriculoperitoneal, ventriculoatrial, and lumboperitoneal shunting [15,16]. Potential complications include obstruction, infection, failure, migration, organ perforation, over or under-drainage, intracranial hemorrhage, seizure, and pseudocyst [16-18]. Patients treated with CSF shunting experience improvement in their symptoms such as gait, cognitive function, and bladder control [19].

The American Academy of Neurology guideline on idiopathic NPH (iNPH) concludes that shunting is possibly effective for patients with idiopathic iNPH with 96% chance of subjective improvement, an 83% chance of improvement on the timed walk test at six months, and an 11% risk of serious adverse events [20]. Among objective measures, only gait improved significantly in the studies included, but in the pooled analysis, 95% did report subjective improvement in symptoms, compared with only 19% of controls. This same guideline also highlights that older age is not associated with a poorer response to shunting, while a good response to lumbar puncture CSF drainage is associated with a good response to shunting.

## Conclusions

Normal pressure hydrocephalus is one of the very few reversible causes of dementia; therefore, early diagnosis and treatment are imperative. Treatment consists of shunting the CSF away from the brain; shunts can be ventriculoperitoneal, ventriculoatrial, or lumboperitoneal. Unfortunately, NPH remains underdiagnosed and thus undertreated, an important reminder that this case provides.
